# Extracellular Vesicles in Cardiac Repair Approaches: Implications for In Vitro Heart Models and Potential ATMP Development

**DOI:** 10.3390/cells15100900

**Published:** 2026-05-14

**Authors:** Simona Di Stefani, Maura Cimino, Rosaria Tinnirello, Martina Maria Cocco, Cinzia Maria Chinnici, Giandomenico Amico, Valentina Di Felice, Filippo Macaluso, Bruno Douradinha, Paolo Di Nardo, Gioacchin Iannolo

**Affiliations:** 1 IRCCS ISMETT (Istituto Mediterraneo per i Trapianti e Terapie ad alta specializzazione), Via E. Tricomi 5, 90127 Palermo, Italy; sdistefani@ismett.edu (S.D.S.); mcimino@ismett.edu (M.C.); rtinnirello@ismett.edu (R.T.); mcocco@ismett.edu (M.M.C.); 2 Department of Biomedicine, Neuroscience and Advanced Diagnostics, University of Palermo, 90133 Palermo, Italy; valentina.difelice@unipa.it (V.D.F.); filippo.macaluso@unipa.it (F.M.); 3 Regenerative Medicine Unit, Ri.MED Foundation, 90133 Palermo, Italy; cchinnici@fondazionerimed.com (C.M.C.); gamico@fondazionerimed.com (G.A.); 4 OneCapsule, Marousi, 15126 Athens, Greece; 5 Interdepartmental Center for Regenerative Medicine (CIMER), University of Rome “Tor Vergata”, 00133 Rome, Italy; dinardo@uniroma2.it; 6 Department of Medicine and Surgery, Università degli Studi di Enna “Kore”, 94100 Enna, Italy

**Keywords:** advanced therapy medicinal products (ATMPs), mesenchymal stromal cells (MSCs), organoids, cardiac progenitor cells, cardiospheres, cardiosphere-derived cells (CDCs), organ-on-a-chip systems, heart-on-a-chip technologies, organ failure, heart transplantation

## Abstract

**Highlights:**

Cardiovascular diseases are one of the most significant challenges in modern medicine. Despite growing interest in this field, important limitations remain in the evaluation of novel therapeutic approaches. In particular, in vivo systems, including animal models, currently represent the closest approximation to clinical conditions for assessing treatment efficacy and safety. However, these approaches are associated with substantial limitations, primarily related to high costs and ethical concerns. Consequently, many studies rely on in vitro models.
Traditional in vitro methods, even when based on primary cells or progenitor cells, suffer from intrinsic limitations in reproducing the structural and functional complexity of cardiac tissue. To address these challenges, advanced in vitro systems known as organoids have recently been developed to better recapitulate the complexity of the organ microenvironment. These systems have increasingly been used to evaluate innovative therapeutic strategies, including extracellular vesicle-based treatments.

**Abstract:**

Cardiovascular diseases remain the leading cause of mortality in developed countries. Among these conditions, acute myocardial infarction (AMI) is associated with particularly high rates of cardiac morbidity and mortality. Cardiac development in mammals is primarily dependent on cardiomyocyte (CM) proliferation during embryonic and early postnatal stages. However, following birth, the proliferative capacity of CMs declines markedly, with only limited cellular renewal occurring during adult life in response to pathological injury. Consequently, the irreversible loss of functional cardiomyocytes and the subsequent formation of fibrotic scar tissue frequently lead to persistent cardiac dysfunction and progressive impairment of cardiac physiology. Cardiomyocyte self-renewal is a tightly regulated process involving multiple molecular pathways. Among factors implicated in this regulation, microRNAs (miRNAs) have emerged as key modulators coordinating both cardiac development and tissue repair mechanisms. In this context, extracellular vesicles (EVs) have attracted considerable interest as potential modulators of these regenerative processes. In particular, mesenchymal stromal cells (MSCs) represent a promising therapeutic platform due to their immunomodulatory and anti-fibrotic properties demonstrated across multiple in vitro and in vivo models. Furthermore, the therapeutic potential of MSC-derived EVs can be enhanced through bioengineering approaches aimed at improving targeted molecular delivery. In this review, we summarize recent advances in the development and application of EV-based therapeutic strategies, with particular emphasis on their potential use as advanced therapy medicinal products (ATMPs) for cardiovascular regeneration and repair.

## 1. Introduction

A growing number of heart disorders associated with an increase in life expectancy in modern countries is a striking health problem worldwide. The economic impact of such cases requires prompt improvement in the quality of life and organ re-establishment after damage [1,2]. Heart transplantation involves from many difficulties, which range from the scarcity of organs to the status of the collected organs. In particular, the processes of explantation, transportation, and reimplantation cause damage in many cases, leading to organs becoming unsuitable for transplantation [3]. Therapeutic approaches that support organ wellness are a key element for long-term successful patient recovery. In this scenario, it appears crucial to investigate new strategies to ameliorate heart function both in vitro and in vivo.

The most reliable model for evaluating cardiac in vitro treatments and ischemic rescue is now represented by cardiac organoids [4]. These in vitro models aim to recapitulate, in vitro, the complexity of the heart architecture, including tissue variability and the structural spatial organization. The greatest value of this system is in reducing the need for in vivo evaluation in preclinical studies, offering a further advantage of cost reduction, in particular concerning the number of animals required. Organoids consist of three-dimensional (3D) cellular structures organized in a complex differentiated structure originating from stem cells, generally iPSCs (induced pluripotent stem cells), which have been induced into a specific commitment to mimic the physiological arrangement in the organ. Their use as an in vitro model over the last two decades has been widely increased. This in vitro model represents the most reliable method for evaluating treatments or drugs. In particular, heart organoids have been improved, creating physiologically active structures. Their composition and structure allow for experimental treatments that resemble the organization of a real body. Their structure can be used to evaluate the stability of cell viability in organ transplantation. Moreover, drugs or physical treatments can be analyzed to improve organ efficiency during explantation or transport. On the other hand, myocardial injury (MI) recovery can be evaluated using in vitro beating hearts, improving the apoptotic damage and treatment rescue. In this regard, real-time monitoring has been proposed using the help of an “organ-on-a-chip” system. In silico treatment simulations on organoids represent an interrelation between cell biology and engineering. This method has recently been proposed as an improved artificial reality to monitor rescue phenomena after physical or chemical injuries.

## 2. Extracellular Vesicles: Characteristics, Isolation, and Therapeutic Implications

Intercellular communication is a fundamental principle in mammalian development and preservation of homeostasis, enabling cells to coordinate their behavior through tightly regulated signaling networks.

Beyond classical mechanisms such as autocrine, paracrine, and endocrine signaling, cells also communicate through the release of EVs. EVs have recently emerged as an attractive tool for cell-free therapy. According to their size, EVs can be categorized into exosomes (30–150 nm), microparticles (100–1000 nm), and apoptotic bodies (500–5000 nm) [5]. EVs encapsulate proteins, lipids, and nucleic acids within a lipid bilayer membrane, enabling their transfer to recipient cells over both short and long distances. This shielding preserves cargo molecular integrity by preventing exposure to proteases, nucleases, and other destabilizing factors, while also reducing immune recognition. As a result, EVs ensure efficient delivery and facilitate cargo transfer to recipient cells over both short and long distances. EVs can be isolated from body fluids [6] or from cells cultured in vitro using several techniques, with the choice of method influencing the yield and purity of the isolated vesicles. The most widely used approach is ultracentrifugation or density gradient centrifugation, and other methods include size exclusion chromatography, immunoaffinity, ultrafiltration, microfluidic technologies, and commercially available kits (columns and resins) [7]. Once isolated, EVs can be characterized using dynamic light scattering, nanoparticle tracking analysis, flow cytometry, label-free nonlinear microscopy approaches, transmission electron microscopy, atomic force microscopy, combined reflectance and fluorescence, confocal microscopy, or Western blotting and ELISA [8].

EVs can be engineered, stored, and standardized [9] more easily than whole cells, making them a promising and versatile platform for regenerative and translational medicine applications.

Depending on the therapeutic goal and the specific type of cardiovascular injury, EVs can be modified with therapeutic messenger RNA (mRNA) [10], miRNAs [11], and other non-coding RNAs [12], which can profoundly influence the post-injury microenvironment by influencing apoptosis, inflammation, angiogenesis, and extracellular matrix remodeling.

Harnessing EVs to deliver therapeutic mRNA is an emerging gene therapy strategy for ischemic heart disease and MI. A key proof of concept comes from a study using EVs loaded with vascular endothelial growth factor A (VEGF-A) mRNA to enhance angiogenesis and functional recovery in ischemic models [13]. In murine myocardial and limb ischemia, EV-mediated delivery of full-length VEGF-A mRNA induced rapid, localized protein expression, resulting in increased neovascularization, improved perfusion, and superior cardiac function compared with saline, lipid nanoparticles, or adeno-associated viral vectors [13]. Notably, repeated EV administration did not trigger significant immune responses, underscoring the favorable safety profile of EV-based mRNA delivery [13].

**Figure 1:**
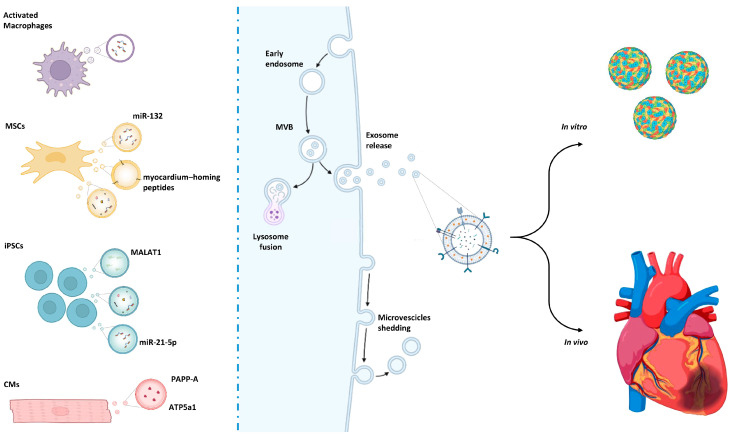
Overview of extracellular vesicle biogenesis and release from various sources. Their application as cargos can offer a valid therapeutic approach for 3D organoids/progenitors or for in vivo treatments.

Beyond promoting neovascularization, miRNA encapsulated in EVs can also modulate inflammatory responses and stimulate endogenous cardiac repair mechanisms [14]. In fact, EVs isolated from various progenitor or stem cells carry endogenous miRNAs, such as miR-126 and miR-146a, which regulate angiogenesis and cell migration and can be further optimized by combining with delivery matrices to enhance retention and therapeutic efficacy in MI models [15,16]. EVs isolated by other sources, such as iPS-derived cardiac progenitor cells, carry a repertoire of enriched miRNAs (miR-302, miR-367, miR-371, miR-372, miR-373, miR-512, miR-520, and miR-548) that reduce post-infarction fibrosis and promote angiogenesis, contributing to improved myocardial remodeling [17]. In particular, one study focused on miR-373’s ability to promote the ability to improve cardiac function [17] in left anterior descending (LAD) ligated mice models. EV engineering was explored in HEK293T cells, which were genetically modified to deliver miR-21-loaded EVs. The exogenous miRNA-21 significantly enhanced survival and angiogenesis in a preclinical MI animal model, resulting in improved functional recovery post-MI (Figure 1). Another study found that miR-21 acts by inhibiting apoptosis and activating pro-angiogenic signaling pathways, such as PTEN/Akt/VEGF in the recipient cardiac tissue and endothelial cells [18].

Despite the ability of HEK293T to be used as a recipient for engineering EVs, MSCs are one of the preferred targets for EV production due to their intrinsic characteristics of imunogencity. miR-132 has been found to mimic loaded EVs (MSC-derived) and promote endothelial tube formation and neovascularization in ischemic hearts of mice [19] (Figure 1). The delivery of miR-132 via EV electroporation enhanced angiogenesis and preserved cardiac function by suppressing target gene RASA1 in endothelial cells [19] (Figure 1).

Recently, targeted siRNA delivery to injured myocardium has emerged as an innovative approach for enhancing cardiac repair by silencing pathogenic genes involved in fibrosis, hypertrophy, or adverse remodeling. LNA34 antisense (Ant34) showed a positive effect in vitro on human cardiac progenitors [20] and in vivo on MI mice models [21].

Modification of EVs has been extended to their surface. A recent study developed a targeted EV platform by decorating human serum-derived EVs with a fibroblast activation protein (FAP) aptamer to achieve preferential delivery of siRNA against TGFβ1 (siTGFβ1) to injured cardiac tissue [22]. Upon systemic administration in angiotensin II-treated mice, these EVs loaded with siTGFβ1 effectively downregulated TGFβ1 in the myocardium, reduced fibrosis, decreased CM hypertrophy, and improved cardiac function without systemic toxicity [22]. Similarly, other studies focused on the ability to precisely engineer their lipid bilayer to enhance cardiac targeting, either through genetic modification of parental cells or via post-isolation surface functionalization of vesicles [23]. In particular, EVs engineered to express high levels of a cardiac-targeting peptide were shown to efficiently deliver siRNA against the receptor for advanced glycation end products (RAGE) in a mouse model of myocarditis. EVs loaded with siRAGE markedly increased cardiac uptake of siRNA compared to unmodified EVs and attenuated myocardial inflammation [23]. For example, a CM-targeting peptide (CTP) was genetically fused to an exosomal membrane protein (Lamp2b), allowing its display on the EV surface isolated from human cardiosphere-derived cells [24]. This strategy significantly increased EV uptake by CMs both in vitro and in murine models of MI, leading to enhanced retention within the infarcted myocardium and improved functional recovery [24]. Wang et al. described the use of ischemic myocardium homing peptides identified through phage display screening. These peptides were conjugated to MSC-EV membranes, improving selective accumulation in the injured heart after systemic administration (Figure 1). Biodistribution analyses have demonstrated reduced off-target uptake (e.g., liver and spleen) compared with unmodified EVs, supporting the specificity of the targeting approach [25].

More recently, advanced conjugation techniques were employed to achieve stable and controlled peptide display on the EVs’ surface. By extrusion, platelet-mimetic EVs (P-EVs) were fabricated by fusing the membranes of MSC-EVs with platelet membranes. These P-EVs promoted angiogesis and gave an additional functional benefit to myocardial ischemia–reperfusion (MI/R) mice models [26].

Collectively, these reports reveal that rational engineering of the EV lipid bilayer with cardiac-homing peptides can increase myocardial targeting efficiency, improving cargo delivery and enhancing therapeutic efficacy in preclinical models of cardiovascular disease (Figure 1).

Therapeutic potential of EVs in cardiac repair and regeneration is closely linked to their cellular origin, which determines their molecular cargo and functional impact in recipient tissues. EVs can originate from multiple cellular sources, most prominently MSCs [27]), but also immune cells [28,29], pluripotent stem cells [30,31,32], tissue-specific progenitors [33,34]), and cardiac cells [35].

As regards immune cell-derived EVs, a recent study showed that the administration of macrophage-derived EVs effectively reduced iron overload in hypoxia-treated CMs and hearts post-MI [36]. In a murine model of myocardial infarction, exosomes derived from M2-polarized macrophages significantly improved cardiac function, reduced infarct size, and enhanced angiogenesis. These therapeutic effects were attributed to the transfer of miR-132-3p to endothelial cells, where it promoted pro-angiogenic signaling and vascular remodeling, thereby contributing to post-ischemic cardiac repair [37]. It was further demonstrated that M2 macrophage-derived exosomes enhance cardiac function and reduce fibrosis after MI by promoting angiogenesis and transferring anti-inflammatory miRNAs to cardiac cells [38]. In contrast, M1 macrophage-derived EVs can exacerbate adverse remodeling, highlighting phenotype-dependent effects of macrophage EVs [39]. On the other hand, regulatory T cell-derived EVs contribute to repair by suppressing excessive inflammation and promoting reparative macrophage polarization, leading to improved ventricular function [40].

Several original preclinical studies have demonstrated that EVs derived from iPSCs or their cardiovascular derivatives exert therapeutic effects in models of MI. In a seminal study, EVs isolated from murine iPSCs improved left ventricular function after MI in mice, reduced CM apoptosis and hypertrophy, and enhanced angiogenesis, while avoiding the tumorigenic risks associated with direct iPSC transplantation [41]. These findings established the concept of iPSC-derived EVs as a safer cell-free alternative for cardiac repair. Subsequently, Santoso et al. [42] showed that exosomes derived from human iPSC-CMs (hiPSC-CMs) promoted myocardial recovery in vivo by activating protective autophagy pathways, leading to reduced fibrosis and apoptosis. This study highlighted the importance of EV cargo, particularly miRNA-mediated signaling, in modulating cardiomyocyte survival. Wu et al. [43] further demonstrated that EVs from human embryonic stem cell-derived cardiovascular progenitors improved infarct healing by enhancing angiogenesis and CM survival, partly through transfer of regulatory long non-coding RNAs (lncRNAs), such as MALAT1 (Figure 1). These data suggest that lineage-committed derivatives of pluripotent stem cells may produce EVs with enhanced cardiac specificity. More recently, engineered or condition-optimized EVs have shown enhanced efficacy. For example, hypoxia-conditioned hiPSC-derived EVs displayed increased cardioprotective activity via activation of pro-survival signaling pathways [44], while miR-21-5p-enriched exosomes from hiPSC-CMs demonstrated superior functional recovery compared to non-enriched EVs [45] (Figure 1). EVs secreted by cardiac progenitor cells (CPCs) reduced CM apoptosis, enhanced endothelial tube formation, and improved left ventricular function in murine myocardial infarction models, in part through the transfer of miRNAs, such as miR-210 and miR-132, which regulate survival and angiogenic pathways (e.g., ephrin A3, RasGAP p120) [46] (Figure 1). The comparative analysis of CPC-released EVs vs. BMC-derived ones indicates a higher cardioprotective effect for the cardiac counterpart. This was attributed to the surface expression of the pregnancy-associated plasma protein-A (PAPP-A) [47] (Figure 1). This effect, evaluated in rat in vivo models, was attributed to the IGF-1 receptor activation and the downstream Akt pathway, with a reduction in caspase activity after artery ligation [47].

EVs produced by cardiac resident cells have also been shown to contribute to heart repair by modulating intercellular signaling in injured myocardium. CMs and other cardiac cell types inherently release EVs that accumulate in ischemic myocardium and participate in repair-related signaling. In fact, EVs from stressed CMs can interact with bone marrow monocytes via chemokine receptors to mobilize progenitor cells and support tissue repair post acute myocardial infarction [48]. Recent experimental work further indicates that EVs directly derived from heart tissue can attenuate ischemia–reperfusion injury by preserving mitochondrial function and reducing ferroptosis through the delivery of mitochondrial proteins such as ATP5a1, highlighting the therapeutic potential of cardiac tissue-derived EVs in ameliorating myocardial damage [49]. CM-derived exosomes generated during ischemia were found to enhance endothelial cell migration and tube formation, indicating that stress-induced vesicular signaling from CMs may contribute to post-ischemic vascular remodeling and cardiac repair [50,51].

MSC-derived EVs are the most extensively investigated due to their immunomodulatory and regenerative properties, which largely recapitulate the paracrine effects of the parental cells without risks associated with live cell transplantation, such as uncontrolled cell proliferation, tumorigenicity, or poor engraftment. Biological effects of MSC-derived EVs are highly context-dependent. Their impact is determined not only by the molecular cargo, but also by the signaling pathways engaged in the recipient cells. In particular, the miRNA content can elicit distinct responses depending on the activation state of the target cells. For example, EVs from MSCs can trigger regenerative programs in injured cells, promoting dedifferentiation, cell cycle re-entry, proliferation, and enhanced survival [52]. In contrast, the same EVs can influence cancer cells differently by delivering tumor-suppressive miRNAs that inhibit proliferation and induce apoptosis [53].

MSC-EVs exerted multifaceted cardioprotective actions across numerous models of cardiovascular diseases. They achieve this by suppressing pathological processes such as pyroptosis [54,55,56], ferroptosis [57,58,59], and excessive autophagy [60,61,62], while modulating intracellular oxidative stress [63,64,65]. As a result, MSC-EVs reduce the secretion of pro-inflammatory cytokines, promote microvascular regeneration, and inhibit apoptosis of cardiomycytes [66]. This ultimately improves cardiac function and tissue recovery following heart damage [67]; particularly, MSC-EVs carrying specific miRNAs provide anti-apoptotic, anti-inflammatory, anti-fibrotic, and angiogenic effects within the infarcted heart [68]. MSC EVs reduce CM apoptosis and attenuated inflammatory responses after ischemic injury, in part by modulating macrophage polarization and downregulating pro-inflammatory cytokines. Preconditioning strategies (such as atorvastatin treatment of MSCs) further enhanced these immunomodulatory and reparative properties of EVs [69]. Moreover, MSC EVs have been shown to enhance neovascularization in infarcted hearts, contributing to improved perfusion and left ventricular function [70]. For example, delivery of bone marrow-derived MSC-EVs restored cardiac function in MI models by increasing vascular density, an effect linked to EV-mediated transfer of pro-angiogenic microRNAs such as miR-210 [71]. H. Cheng et al. found that MSC-derived exosomes enriched in miR-210 reduced infarct size and improved cardiac function after coronary ligation in rats. A direct cardiac injection of these exosomes enhanced CM survival under hypoxic conditions and reduced CM apoptosis, with improved functional outcomes in vivo [72]. Zheng et al. showed that exosomes from bone marrow MSCs deliver miR-29b 3p in a rat MI model, which improved myocardial angiogenesis, reduced fibrosis, enhanced VEGF expression, increased capillary density, suppressed CM apoptosis, and improved ventricular remodeling and function [73].

Overall, MSC-derived EVs indicate broad cardioprotective potential across various cardiovascular diseases by modulating cell survival, inflammation, oxidative stress, autophagy, and vascular remodeling, thereby supporting improved cardiac structure and function. By targeting multiple pathways simultaneously, MSC EVs ameliorate myocardial structure, vascularization, and cardiac function without risks associated with direct stem cell transplantation. Their intrinsic biocompatibility, low immunogenicity, and potential for engineering make MSC EVs a versatile and attractive platform for developing regenerative therapies in cardiovascular medicine (Figure 1).

Given their capacity to exert pleiotropic effects across multiple biological pathways, EVs are a potential therapeutic strategy for cardiovascular diseases. Consequently, EVs are currently being investigated to evaluate their safety and therapeutic potential. However, clinical studies evaluating EVs for cardiac indications are still in early stages, and current evidence for their use in patients is still limited. At the time of writing, only three clinical trials have focused on administration of EVs for cardiovascular disease treatment. One of them, NCT05669144, is evaluating the administration of UC-MSC-derived EVs in patients undergoing coronary artery bypass grafting after MI. The novelty of this study resides in the co-administration of autologous mitochondria isolated from MSCs in combination and alone to evaluate possible improvements in the patient’s condition after MI. Another trial, NCT05774509, assesses the safety and efficacy of three intravenous injections of extracellular vesicle-enriched secretome of cardiovascular progenitor cells in severely symptomatic patients with drug refractory left ventricular (LV) dysfunction secondary to non-ischemic dilated cardiomyopathy. The evaluation of treated patients was fixed at 12 months after injection and was based on changes in LV function, oxygen consumption, plasmatic levels of natriuretic peptide (BNP or NT-ProBNP), and, more importantly, serious adverse effects that indicate the safety of this procedure. Among others, the only completed study was done by evaluating intracoronary infusion of EVs in patients following coronary stent implantation (NCT04327635). This study aimed to evaluate dose-limiting toxicities (DLTs) and maximum tolerated dose (MTD) of a single administration (10 mL) of purified exosome product (PEP) harvested from human apheresis blood [74] at escalating concentrations of extracellular vesicles delivered at a single time point. The outcome parameters post-intervention were focused on the evaluation of infarction scar size, the ejection fraction, and the alloimmune response. All described trials are pioneering in this field, and their focus has been on safety, feasibility, and preliminary efficacy. In this regard, it must be considered that one of the most critical concerns in the use of EVs as therapeutic agents is focused on safety. In particular, the rules defined by the Food and Drug Administration (FDA) [75,76], the European Medicines Agency (EMA) [77], and the International Society for Extracellular Vesicles (ISEV) [78] attempt to fix the requirements for EVs as therapeutic agents in a similar way to standard drugs. Further studies are needed to establish clinical efficacy, optimal dosing, and long-term outcomes, with a validation in well-designed clinical trials before translation into routine cardiovascular care.

## 3. Cardiac Progenitors and Cardiac Organoids

As outlined above, EVs represent an interesting therapeutic approach for evaluating MI interventions. However, it seems appropriate to seek a faithful model to test the effects through in vitro simulation. One of the most diffuse and reliable in vitro models for heart study and the analysis of the mechanisms involved in repair is represented by CMs. These cells exhibit a pronounced proliferative capacity that supports cardiac growth during development and show high regenerative potential. This ability rapidly diminishes during the early postnatal period, where CMs progressively exit from the cell cycle and undergo terminal maturation [79]. Consequently, adult mammalian heart displays a limited ability to replace lost CMs after injury. Despite the fact that adult CMs retain a minimal potential to re-enter the cell cycle, this residual turnover remains insufficient to achieve functional myocardial regeneration [80]. Within this context, the maintenance of a low level of CM renewal may be supported by a resident population of CPCs [81]. CPCs are believed to reside in specialized niches within distinct regions of the heart, including atrial domains and the outflow tract. These cells can be isolated from several cardiac compartments, such as epicardial, myocardial, and perivascular tissues [82]. Functionally, CPCs exhibit stemness properties, including self-renewal and multilineage differentiation capacity [83]. Because of their multipotent nature, these cells are able to differentiate into the main cardiac cell types, including CMs, smooth muscle cells, and endothelial cells [84]. Over the years, diverse populations of cardiac progenitor cells have been described, originating from different anatomical regions of the heart and from various developmental stages. Their identification and classification have largely relied on the analysis of specific surface antigens and lineage-associated transcriptional signatures, which are used to distinguish functionally and phenotypically distinct subsets within the broader CPC compartment [83]. The identification of resident cardiac stem/progenitor cells dates back to 2003, when a population isolated from the adult rat myocardium was described on the basis of c-Kit expression and the absence of hematopoietic lineage markers. This cell fraction expressed early cardiac transcription factors, including Nkx2.5, GATA4, and MEF2C [83]. Functional analyses indicated that the Lin−/c-Kit+ population fulfilled the principal criteria attributed to stem cells, demonstrating sustained proliferative capacity, clonogenicity, and differentiation into multiple cardiovascular lineages. In particular, these cells were reported to differentiate into CMs, vascular smooth muscle cells, and endothelial cells, supporting their proposed multipotent profile. Evaluation of these cells in transplantation experiments demonstrated their regenerative potential. Administration of these cells, or of clonally derived progeny, into ischemic myocardium was associated with the formation of newly organized myocardial tissues, restoring a substantial portion of the left ventricular wall. The regenerated region included newly formed vasculature and CMs that, though smaller than fully mature counterparts, displayed structural organization and functional properties consistent with developing myocardial cells [85]. Genetic lineage-tracing analyses conducted by van Berlo et al. indicated that c-Kit+ cells contribute only marginally to the generation of new CMs in the in vivo adult heart [86]. In addition to this population, another group of cardiac progenitor cells present in the adult myocardium is characterized by the expression of stem cell antigen-1 (Sca-1).

In vitro studies have also shown that Bmi1+ cells are capable of differentiating into cell types with properties similar to both CMs and vascular smooth muscle cells [87]. Importantly, Sca-1 is a marker restricted to mice, with no identified human equivalent to date [88]. Beyond the Sca-1+ lineage, side population (SP) cells constitute another category of heart resident progenitors. These cells possess the ability to generate CMs, smooth muscle cells, and endothelial cells [89,90]. Mouse transplantation experiments indicate that SP cells can home damaged myocardial regions and integrate into existing cardiac tissue, suggesting their potential as candidates for regenerative interventions following cardiac injury [90].

Among the assessments for in vitro cardiac models to evaluate innovative treatments, CPCs and cardiosphere-derived cells (CDCs) have focused considerable attention in the field of cardiac regeneration. Their identification as cardiac precursor cells, which are able to colonize the injured heart and able to differentiate, contributing to organ repair, makes them an attractive model for studying therapeutic approaches [91,92]. These cells originate from three-dimensional multicellular structures known as cardiospheres, which are in turn generated from human myocardial tissue samples obtained from atrial or ventricular biopsies. For their isolation, small fragments of cardiac tissue are initially maintained under culture conditions that promote cell migration from the explant. The migrating cells are subsequently cultured in conditions that favor 3D aggregation, leading to the cardiosphere formation [82]. Cardiospheres recapitulate a microenvironment resembling a stem cell niche, characterized by the presence of multiple interacting cell types organized within a three-dimensional architecture. Within these structures, c-Kit^+^ cardiac stem cells are predominantly localized in the central core of the spheroid, where they are surrounded by accessory-supporting cell populations, including mesenchymal, cardiomyogenic, and vasculogenic cells, preferentially distributed along the outer layers of the cluster. These cellular components remain closely associated through interactions mediated by extracellular matrix molecules [93]. Compared with cardiosphere-forming cells maintained under conventional two-dimensional culture conditions, cells organized in a three-dimensional structure display a distinct molecular profile. In particular, they exhibit increased expression of vascular progenitor markers, such as PDGFRα, c-Kit, and VEGFR1, together with elevated levels of genes involved in angiogenesis regulation, including VEGF, FGF2, and ANGPT1. Furthermore, cardiospheres show enhanced secretion of several pro-angiogenic mediators, such as CXCL16, and PlGF-2, in addition to the already mentioned ANGPT1 and VEGF. The production of these factors is thought to promote reparative angiogenic responses, a key component of the processes underlying cardiac repair and regeneration [94]. The dissociation of cardiospheres and their subsequent culture under adherent conditions enables the establishment of an expandable monolayer population of CDCs [82]. These cells are characterized by the ability to generate colonies, maintain self-renewal, and differentiate into various cardiovascular cell types. Even when derived from single clones, these populations show intrinsic phenotypic variability. During in vitro expansion, a minor fraction preserves progenitor-like characteristics, whereas other cells gradually progress toward more specialized states, including CM, smooth muscle, and endothelial phenotypes, which reflects the intrinsic multipotent nature of these cells [95]. miRNA profiling studies have identified a distinct signature enriched in CSs/CDCs. Several of these miRNAs appear to orchestrate the proliferative potential of progenitors while maintaining their undifferentiated phenotype. In particular, miR-31 is markedly upregulated in undifferentiated progenitors [96], consistent with its well-established role in regulating cellular migration and proliferation through multiple molecular targets [97]. Moreover miR-21 is strongly expressed in progenitor populations [96] and has been extensively characterized as a key mediator of cardioprotective signaling. Experimental models of myocardial infarction have demonstrated that conditioned media derived from CMs can attenuate infarct size via miR-21-dependent mechanisms, underscoring its ability to transmit protective paracrine signals [98]. Over the past decade, CDCs have been extensively studied for their therapeutic potential in ischemic heart disease, demonstrating beneficial effects across multiple experimental models of MI. The reparative activity of cardiosphere-derived cells has been attributed to two main mechanisms. One involves their ability to differentiate into CM-like cells, though this contribution appears relatively minor. Increasing evidence suggests that the primary mechanism is paracrine in nature, mediated by the release of bioactive factors within the CDC secretome that modulate the cardiac microenvironment and promote tissue repair [99]. Analyses of conditioned media from human cardiospheres and CDCs have revealed that these cardiac progenitor populations release a broad repertoire of soluble mediators with reparative potential. Among the most relevant factors are the VEGF, HGF, and IGF-1 [99] molecules, all widely recognized for their involvement in the response to ischemic MI [100,101,102,103,104,105,106]. These trophic signals have been associated with multiple beneficial effects, including suppression of CM apoptosis, improvement of microvascular perfusion, and attenuation of adverse ventricular remodeling following myocardial infarction [100,101,102,103,104,105,106]. In vitro, exposure of neonatal rat ventricular myocytes to CDC-conditioned medium significantly reduces early apoptosis, highlighting a pronounced cytoprotective activity. Moreover, the CDC secretome exhibits marked pro-angiogenic properties, as exposure of human endothelial cells to CDC-derived conditioned media markedly enhances their ability to form complex capillary-like tubular networks, suggesting a stimulation of neovascularization. Consistently, in vivo administration of CDCs in a SCID mouse model of acute myocardial infarction (AMI) has been associated with activation of survival-related signaling pathways and reduced expression of apoptotic markers. These observations support the concept that paracrine mediators released by CDCs contribute substantially to myocardial protection and tissue repair after ischemic injury [99]. Among the components of the CDC secretome, EVs have recently emerged as key mediators of these paracrine effects. Studies performed in porcine models of myocardial infarction have shown that EVs released by CDCs (CDC-EVs) can modulate the post-infarction inflammatory response by promoting macrophage polarization toward the anti-inflammatory and pro-reparative M2 phenotype, thereby limiting the excessive inflammatory activation characterizing the early phase of cardiac injury [107]. This macrophage reprogramming is associated with enhanced endothelial cell migration, early activation of cardiac fibroblasts, and increased extracellular matrix turnover, processes that collectively contribute to tissue repair following ischemic damage. These effects have been linked to a cytokine milieu enriched in pro-angiogenic and reparative mediators, including VEGF-A, TGFB1, TGFB2, CXCL14, and IL33, which together support the establishment of a microenvironment favorable for cardiac remodeling and functional recovery [108]. The clinical potential of CDCs has been evaluated in patients with myocardial infarction in the CArdiosphere-Derived aUtologous stem CElls to reverse ventricUlar dysfunction (CADUCEUS) trial, a prospective randomized phase I study. In this trial, intracoronary infusion of autologous CDCs demonstrated a favorable safety profile and was associated with structural improvements in the infarcted myocardium. In particular, cardiac magnetic resonance imaging performed at six months revealed a significant reduction in scar mass together with an increase in viable myocardial tissue, accompanied by improvements in regional contractility and systolic wall thickening [91].

While CDCs have demonstrated significant regenerative potential in preclinical and clinical studies, replicating the structural and cellular complexity of the myocardium in vivo remains challenging. Silk fibroin-based scaffolds cellularized with c-Kit+ cardiac progenitor cells (CPCs) embedded in type I collagen have been engineered to generate functional, vascularized cardiac constructs. These systems supported cellular proliferation and the maintenance of cardiac differentiation markers in vitro. However, in vivo implantation in immunodeficient mice triggered host immune responses, including expansion of CD3^+^ T lymphocytes [109].

These findings emphasize the need for advanced tissue models. In this scenario, the generation of 3D structures, such as cardiac organoids, has recently been achieved to recapitulate more faithfully the cellular and functional complexity of the myocardium. Cardiac organoids are 3D multicellular systems that arise through the intrinsic self-organization of cardiac progenitor cells and their differentiated derivatives within a structured microenvironment. These tissue-like miniature constructs typically include CMs together with supporting cell populations, such as endothelial cells and cardiac fibroblasts, thereby partially reproducing the cellular diversity of native myocardial tissue [110,111].

Over the years, several strategies have been developed to generate cardiac organoids in vitro using pluripotent stem cells, cardiac progenitors, or differentiated cardiac cell types. Broadly, these approaches can be categorized into scaffold-based and scaffold-free systems. Scaffold-based methods employ biomaterial matrices, such as hydrogels or decellularized extracellular matrices, which provide structural support and biochemical cues that facilitate tissue organization. In contrast, scaffold-free approaches rely on the spontaneous aggregation and self-assembly of cells under low-adhesion culture conditions, allowing the formation of spheroidal 3D structures. Additional bioengineering technologies have further expanded the range of available models, including microfabrication platforms and 3D bioprinting. In these strategies, the establishment of a 3D architecture is crucial for maintaining cellular morphology, phenotypic identity, and ensuring proper cell polarity, while also promoting physiologically relevant cell–cell and cell–matrix interactions [112]. Among the different models developed to date, cardiac organoids derived from human-induced pluripotent stem cells (hiPSCs) have attracted particular interest because of their ability to generate multicellular cardiac structures that resemble the composition of the human heart. In these systems, pluripotent stem cells are first aggregated into embryoid body-like clusters, within which developmental signaling pathways guide the progressive emergence of multiple cardiac lineages [113]. Most current differentiation protocols are based on the biphasic modulation of Wnt signaling [114,115]. Activation of the Wnt pathway at early stages drives the specification of cardiogenic mesoderm and subsequent cardiac lineage commitment. Optimized protocols further incorporate growth factors and pathway modulators, such as BMP, VEGF, FGF, and TGF-β/Smad inhibitors to promote the simultaneous development of CMs, endothelial cells, and fibroblasts, resulting in organoids that more closely approximate adult myocardial structure and function [113].

Comparative analyses of CM differentiation in conventional 2D monolayers and 3D cardiac organoid systems demonstrate that a 3D microenvironment can enhance the maturation of hiPSC-derived CMs. During the early stages of differentiation, cells cultured in 3D structures exhibit increased expression of transcriptional regulators associated with mesoderm induction and cardiac lineage commitment, including MESP1, GATA4, TBX5, and MSX2, suggesting more efficient specification toward the cardiac lineage [116]. CMs derived from organoid cultures display more advanced structural maturation. In particular, the expression pattern of contractile protein isoforms indicates a shift toward an adult-like phenotype, characterized by increased levels of α-myosin heavy chain (MYH6) and cardiac troponin I3 (TNNI3) and reduced expression of fetal-associated isoforms, reflecting progressive organization of the sarcomeric contractile machinery. In addition, organoid-derived CMs exhibit a more mature electrophysiological profile, with an elevated expression of key cardiac ion channel genes together with increased levels of proteins involved in electrical coupling and excitation–contraction processes, including connexin-43 and Cav1.2. These molecular features are associated with improved calcium handling dynamics and more stable contractile activity [116].

Collectively, these features highlight the potential of cardiac organoid systems as advanced experimental platforms for investigating cardiovascular development, modeling cardiac diseases, and performing pharmacological and toxicity screening [117]. hiPSC-derived cardiac organoids, comprising CMs, fibroblasts, and endothelial cells, have emerged as physiologically relevant models for investigating MI and fibrotic remodeling. To emulate ischemic conditions characteristic of AMI, these organoids have been subjected to a hypoxia-mimicking treatment, which resulted in the upregulation of hypoxia-inducible factor-1α (HIF-1α). Under these ischemia–reperfusion (IR) conditions, cardiac organoids displayed a marked increase in apoptotic events, including elevated cleaved caspase-3 and enhanced Bax/Bcl2 signaling. Structural analysis revealed significant disassembly of sarcomeric organization in cardiac organoids following IR injury, accompanied by a reduction in CM-specific markers, such as cTnT and cTnI [83]. Inflammatory and stress-related pathways, including NF-κB activation and phosphorylation of ERK, JNK, and p38, indicative of the engagement of molecular cascades involved in post-infarction cardiac remodeling [118,119], were markedly upregulated, suggesting enhanced tissue remodeling processes [113]. Furthermore, IR-injured cardiac organoids displayed enhanced SERCA activity, resulting in greater calcium uptake into the sarcoplasmic reticulum, a process further supported by increased phosphorylation of phospholamban (PLN) [113].

Collectively, the characterization of cardiac progenitor cells, together with advances in CSs/CDCs expansion and cardiac organoid modeling, has provided a valuable framework for understanding cardiac regenerative potential. These insights also pave the way for emerging therapeutic strategies aimed at promoting cardiac repair, including the extracellular vesicle-mediated delivery of regulatory molecules such as miRNAs, which will be discussed in the following section.

## 4. Organ-on-a-Chip

**Figure 2:**
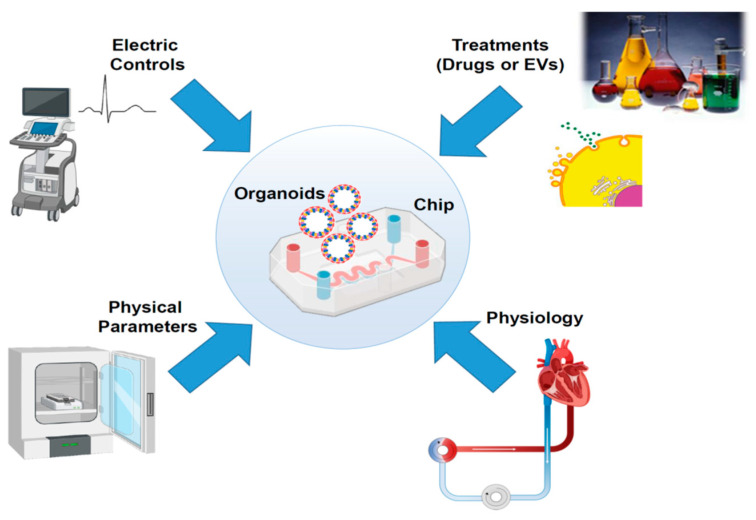
Schematic representation of the integration strategies obtained with the heart-on-a-chip technology: cardiac organoids can mimic ex vivo circulation and be used to evaluate the electrophysiological response. Moreover, the system constitutes a platform for testing drugs or ATMPs under physiological and stress conditions (i.e., hypoxia, temperature, pH).

Despite the significant results in culturing 3D structures such as CSs or organoids, a striking improvement in this methodology was achieved with the implementation of this technique with an in silico control/support: the organ-on-a-chip system. These platforms combine microfluidic elements to reproduce the organ physiology in the human body. The connection between engineering technology and new-generation culture methods, such as 3D organoids, represents an unexplored opportunity to test stress conditions, drugs, and therapeutic interventions, such as ATMPs (Figure 2). The possibility to mimic, in strictly controlled conditions, the ex vivo preservation of the organ destined for transplantation offers an unprecedented venue for cardiovascular applications, supporting in the same way cardiac interventions and transplantation processes. The outcomes obtained in this way result in a more predictive analysis compared to the standard two-dimensional cell culture methods. In the last decade, many groups have proposed innovative solutions to recreate the cardiac microenvironment, which can be finely modulated to test, in vitro, ischemic treatments with consequent fibrotic induction and/or apoptotic cascade. The first models of chips have been used in CM monolayer cell cultures [120,121]. These elements offer the opportunity to control fluidic exchange and electrophysiological properties under stimulation, evaluating cell contractility and allowing pharmacological studies and chemical compound library screening [120,121]. After more than a decade, with the improvement of 3D cell culture techniques, the development of a new system was evaluated, obtaining structures with microcavity arrays (MCAs) through high precision selective laser etching [122]. This system enabled the evaluation of contraction rates of CM clusters after treatment with cardioactive compounds (norepinephrine and blebbistatin) [122]. A similar solution was proposed by another group, integrating a microfluidic chip with a biosensoric approach: MiCAPSUiD (MicroCavitiyArray with Purification and Synthesis-Unit and impedimetric Detection) [123]. The system was tested, as proof of principle, with the administration of propranolol, a cardioactive drug [123]. Using this chip, the authors demonstrated the integration of a quantitative multiparametric analysis of 3D CMs using microfluidic administration of the cardioactive drug propanol in a continuous mode while evaluating cellular bioactivity and the impedimetric magnitude with the biosensors [123]. Another group evaluated the design of a 3D-net-assisted microelectrode array platform [124]. This system is an attempt to enable organoid 3D monitoring in a stable mode. The signal acquisition provides a flexible 3D sensor platform, with a stable contact among organoids and electrodes, preventing floatation and ensuring a minimal volume of culture medium [124]. In this structure, the organoids maintain naive beating while sustaining a stable contact with the electrodes [124]. The evaluation of depolarization/repolarization events in standard conditions and after drug administration is an extraordinary opportunity to test in vitro heart response in real time. One strong advantage in this protocol is the reproducibility of the system by quality control (QC) evaluation and the evaluation of comparable organoid structure in size and in beating efficiency [124]. To improve cellular response reliability in microfluidic chambers, a computational method was recently introduced [125]. This computational fluid dynamics approach (CFD) improved the geometric analysis of the organ-on-a-chip system, increasing the evaluation of cellular response after stimulation [125]. Despite these outstanding systems, preclinical models may not fully capture the complexity of human cardiovascular disease: in particular, the systemic features of these disorders, and the comorbidity factors can cause an imbalance that is hard to replicate with in vivo models.

An improvement in this technological resource would enhance pathophysiological investigation and the evaluation of the potential mechanisms of heart repair in vitro (Figure 2). This would enable the finest evaluation of cardiotoxicity that can support organ recovery not only in vitro, but also in increasing the availability of organs allocated for transplantation.

## 5. Conclusions and Remarks

The increasing demand for high-throughput screening to evaluate drugs and advanced therapeutic approaches in heart repair/recovery requires an enormous effort, in particular in in vivo tests. However, animal model tests are a bottleneck not only from the ethical point of view, but also due to the limits in the animal costs. At the same time, cardiovascular diseases are a major problem for global health management. As a result, it is essential to improve in vitro tests that can be reliable for the study of pathophysiological mechanisms and, at the same time, provide a dynamic model that can recapitulate stress conditions, such as organ transplantation. As described above, 3D organoids and cardiac progenitors provide a strong response to these requests. In particular, the integration of 3D analysis systems, such as chips or scaffolds [126], contributes to the achievement of a reproducible system for evaluating apoptotic induction or fibrotic transformation (Figure 2). In conclusion, heart-on-a-chip systems can fulfill the need for miniaturization, large-scale analysis, and reproducibility at the same time, reducing effective costs.

The analysis of new therapeutic approaches can easily start with these models. For example, it has recently been observed that miRNA-enriched EVs can promote vasculogenesis in self-assembled cardiac tissues on a chip platform [127]. In a similar mode, the role of angiotensin II in a heart-on-a-chip system has been observed, evaluating the response to the SARS-CoV-2 infection [128]. In this case, the system outperformed the in vivo assay for the specificity of viral infection, confirming the potential of an organ-on-a-chip analysis. A future implementation of this system can be achieved by the combination of different organs in the same chip to achieve a better comprehension of the physiological interaction among innovative treatments in a complex in vitro system. This multifaceted configuration can represent a strong approximation of the in vivo preclinical models, greatly improving the prognostic results with respect to the classical 2D cultures. The final result in the design of a novel cell-free therapy is strictly connected to the standardization processes for EV preparation [129]. It has largely evaluated the need for an integrative strategy to merge different sources of EVs (UC-MSC, Wharton’s jelly-derived MSC, dermic MSCs, BM-MSCs) within the isolation method and in the fulfillment of GMP procedure to generate a product that can be available for ATMPs [129,130,131,132,133,134]. A recent report proposed a standardization through a specific signature on the MSC-EV surface [135]. However, it is no easy task to achieve good preparation in a large-scale approach, maintaining GMP features for clinical translation [130,133,134,135]. In place of standard ultracentrifugation methods, tangential flow filtration (TFF) has been proposed. This protocol was proposed as a GMP-grade method for EV preparation for clinical trials and the subsequent use as ATMPs [133,134]. However, despite promising results obtained in preclinical approaches (in vitro and in vivo), translation to clinical practice remains a distant objective. Nonetheless, various reports have tried to fix specific rules in the characterization of all procedures, which must consider batch reproducibility, origin, and different sources of EVs [136]. The European Commission recently defined a document for GMP production [137]. However, it must be considered that cell derivatives do not easily fit with standard drugs, first for batch reproducibility, which can be influenced by culture media and expansion procedures. Moreover, despite the fact that MSCs and their products display low immunogenicity, produced EVs must be evaluated to avoid immune response, as indicated in the trial NCT04327635.

Despite all current difficulties, we expect that in the near future this cell-free system can support therapeutic intervention on MI patients and ameliorate transplantation procedures. 

## Data Availability

No new data were created or analyzed in this study.
